# Prevailing Knowledge on the Bioavailability and Biological Activities of Sulphur Compounds from Alliums: A Potential Drug Candidate

**DOI:** 10.3390/molecules25184111

**Published:** 2020-09-09

**Authors:** Murugan Sesha Subramanian, Giri Nandagopal MS, Syafinaz Amin Nordin, Karuppiah Thilakavathy, Narcisse Joseph

**Affiliations:** 1 Department of Medical Microbiology and Parasitology, Faculty of Medicine and Health Sciences, Universiti Putra Malaysia, Serdang 43400, Malaysia; seshae5@gmail.com (M.S.S.); syafinaz@upm.edu.my (S.A.N.); 2 Department of Mechanical Engineering, Indian Institute of Technology, Kharagpur 721302, India; m.s.girinandagopal@gmail.com; 3 Department of Biomedical Science, Faculty of Medicine and Health Sciences, Universiti Putra Malaysia, Serdang 43400, Malaysia; thilathy@upm.edu.my

**Keywords:** garlic, *Allium sativum*, sulphur compounds, cardiovascular diseases, obesity, cancer

## Abstract

*Allium sativum* (garlic) is widely known and is consumed as a natural prophylactic worldwide. It produces more than 200 identified chemical compounds, with more than 20 different kinds of sulfide compounds. The sulfide compounds particularly are proven to contribute to its various biological roles and pharmacological properties such as antimicrobial, antithrombotic, hypoglycemic, antitumour, and hypolipidemic. Therefore, it is often referred as disease-preventive food. Sulphur-containing compounds from *A. sativum* are derivatives of S-alkenyl-l-cysteine sulfoxides, ajoene molecules, thiosulfinates, sulfides, and S-allylcysteine. This review presents an overview of the water-soluble and oil-soluble sulphur based phytochemical compounds present in garlic, highlighting their mechanism of action in treating various health conditions. However, its role as a therapeutic agent should be extensively studied as it depends on factors such as the effective dosage and the suitable method of preparation.

## 1. Introduction

Garlic (*Allium sativum*) is an ancient civilised plant, originated from the Asian continent between the Mediterranean and China over 600 years ago. Humans use garlic as a medicinal herb in food as well as to relieve from pain and physical and emotional stress. Currently, people are looking for alternative natural medicine. The utilisation of herbs and remedies has brought astounding outcomes to people, and garlic has many medical applications. Garlic products are used as medicine in several ways in day-to-day life activities. Chronic diseases can be treated with very popular health foods associated with natural plant origin. Currently, chronic diseases are the major threat to human health and economic growth, which estimates US$47 trillion for the five major chronic diseases which includes mental illness, diabetes, cancer, chronic respiratory diseases and cardiovascular diseases. The abundance of chemical compounds such as sulphur in garlic has proven its beneficial effects against various diseases. Garlic has the possible positive effect in treating fungal skin infections such as jock itch, ringworm or athlete’s foot.

People consume a capsule of garlic worldwide as it has an antimicrobial effect and can also cure high cholesterol, thrombosis, hyperlipidaemia and Alzheimer’s diseases. Moreover, in India it is used as a galactagogue to enhance lactation in humans. It contains antioxidants that help to destroy the free radicals. Many people use crushed garlic mixed with coconut oil to prevent hair loss and it can promote the regeneration of hair growth. Garlic has been consumed in multiple aspects, as cooked or raw. Garlic has been included in food dishes, which include soups, chutney, salads, fish, and meat curry. The plants of allium have powerful antioxidants, sulphur and phenolic compounds, which serve as the most attractive quality in the food industry. The chemical compounds in garlic have proven its beneficial effects against various diseases including infections, snake bites, hypertension, blood fibrinolytic activity andhyperlipidaemia. Many clinical studies revealed the post effectiveness of garlic towards lowering the cholesterol formation rate, inhibition of enzymes affecting lipid synthesis and angiotensin-converting enzymes. Garlic minimizes the parameters like the low-density lipoprotein (LDL) oxidation rate and reduces the platelet aggregation resulting in decreased blood pressure as it controls the cardiac arrest of a heart patient [1]. The black fresh garlic can raise the immune response with reduced side effects under high temperatures and humidity. Black garlic (BG) was widely used against the treatment of diabetes through the proliferation of monocytes and granulocytes and reduces lymphocytes production rates. BG has the potential beneficial effects on allergic disorders [2]. For many years it is used to treat the diseases like the common cold, illnesses, chronic respiratory diseases, sexually transmitted diseases, wound infection, malaria, cough, mental illness, lung tuberculosis, kidney diseases, liver diseases, asthma, chronic respiratory diseases, and cardiovascular diseases [3]. It has the ability to kill parasites, bacteria, and fungi as well as to protect the liver. Diallyl trisulfide (DATS) from garlic has unique medicinal therapeutic uses, as it inhibits the proliferation of cancerous cell growth through apoptosis and cell cycle arrest [4]. It provides cardiovascular protection, which lowers the cholesterol and blood pressure level. It gives protection against atherosclerosis and helps to decrease the levels of serum glucose, triglycerides, and uric acid as well as insulin resistance and reduces cytokine levels [5].

Generally, garlic can enhance the immune system and exhibits anticancer and chemopreventive activities. It contains active antioxidants involved in cardioprotective and neuroprotective actions. Many preclinical animal model studies showed that the compounds of organosulphur from garlic can inhibit the growth of transplanted as well as spontaneous cancers without any adverse side effects. In addition to this, garlic can greatly act as antioxidant, apoptotic, anti-inflammatory, immunomodulatory, hepatoprotective, anti-invasive and chemopreventive agents [6]. Garlic has proven to exhibit antiviral action against Influenza A and B, cytomegalovirus, rhinovirus, HIV, herpes simplex virus 1 and 2, and rotavirus [2,7,8]. The organosulphur compounds from garlic demonstrate an antimicrobial effect. Even though numerous medicinal plants exist, garlic has a specific antimicrobial property to protect the host from pathogens [9,10,11]. Garlic is more effective with fewer side effects compared to available antibiotics in the market. Though several technical advancements have been achieved in the field of medicine, several diseases remain incurable and fatal for the human race.

Hence, identification and understanding of molecular mechanisms of various diseases are essential to develop novel treatment strategies. In this connection, we have reviewed the therapeutic potential of garlic and its compounds in various fatal disorders. In the review, we focus on various water-soluble and oil-soluble sulphur based phytochemical compounds present in garlic. These sulphur compounds have a key role to play on the pharmaceutical mechanism in treating health conditions. Here, we have highlighted the mechanism of action of various sulphur ingredients in treating cancer, cardiovascular diseases, obesity, immune disorders and obesity. The review will revisit the interesting studies reported on garlic phytochemicals and their pharmaceutical activity.

## 2. Types of Active Compounds

**Figure 1:**
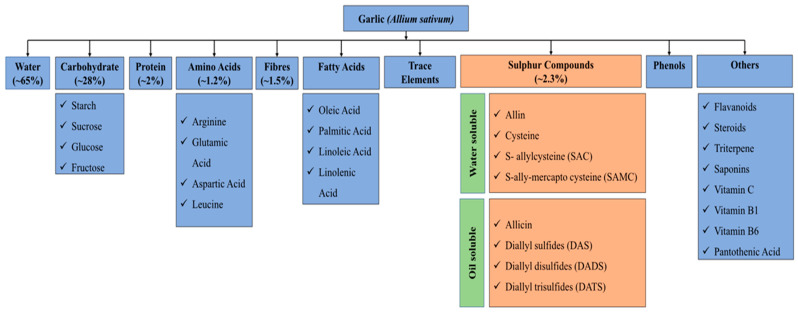
Constituents of garlic *Allium sativum* (Source: adapted from “Phytochemicals of garlic: Promising candidate for cancer” by Zhang et al., 2020. Copyright © Elsevier [16]).

The garlic bulb contains approximately 65% water, 28% carbohydrate, 2% protein, 1.2% amino acids, 1.5% fibres, fatty acids, sulphur compounds, phenols and various other vitamins, iodine, chlorine and various minerals (Figure 1) [12]. The cell cytoplasm of garlic contains S-2-propenyl-l-cysteine S-oxide (alliin). When the garlic was squashed, a catalytic enzyme of alliinase will be released from the vacuole of the cell, which reacts with alliin to form a compound called allicin [13,14].

The dry weight of garlic contains fructose, sulphur, protein, fibres and amino acids of lysine, arginine, histidine, aspartic acid, threonine, glutamine, proline, glycine, cysteine, alanine, valine, methionine, isoleucine, leucine, tryptophan, and phenylalanine. In addition, it contains calcium, protein, iron, zinc, germanium, niacin, riboflavin, carbohydrates, ascorbic acids, folic acids, iodine, pyridoxine, fat, sodium, potassium, saponins, selenium, phosphorous, Vitamin A, Vitamin C, B complex Vitamins, phenols, and manganese. Allyl mercaptan, dipropyl disulphide and allyl methyl sulphide are the water-insoluble components present in garlic. The intact garlic bulbs contain a high amount of γ-glutamylcysteine. Sulphur compounds have a wide range of pharmacological properties and play a key role as an active pharmaceutical component. Alliin, S-allylcysteine sulphoxide, S-allylcysteine and S-allymercapto cysteine are the water-soluble sulphur compounds whereas allicin, diallyl trisulphide, dipropyl sulphide and diallyl disulphide are the oil-soluble sulphur compounds present in garlic [15].

## 3. Sulphur Compounds

There are more than 20 different types of organic sulphur compounds from the few sulphur -containing amino acids produced by garlic and they serve diverse functions. Sulphur compounds in garlic are classified as water-soluble sulphur compounds and oil-soluble sulphur compounds. The structure and chemical composition of the compounds are listed in Table 1.

### 3.1. Water-Soluble Sulphur Compounds

#### 3.1.1. Alliin

Alliin is one of the key water-soluble sulphur compounds in garlic. Alliin forms the stable precursor for the formation of allicin. Alliin catalysed by alliinase form allicin, which is the major bioactive compound in garlic [16,17]. Alliin has demonstrated good antibacterial and anti-malignant properties [18]. Alliin has also shown to have hypoglycemic effect similar to that of glibenclamide [19].

#### 3.1.2. S-Allylcysteine (SAC)

SAC is a bioactive component of the aqueous garlic extract with plenty of established medicinal effects. SAC is known for its actions in the inhibition of Nuclear Factor-kB activation in T cells. SAC is also proved to be effective against inflammatory diseases by the inhibition of inducible nitric oxide synthase expression, along with the maintenance of nitrous oxide produced by endothelial nitric oxide synthase [20]. SAC is also found to affect the proliferation and differentiation of LAN-5 human neuroblastoma cells in vitro [19]. SAC has also been found to exhibit exuberant neuroprotective potential in the rat ischaemia model [21]. Studies of SAC on the antioxidant defence system of the pancreas in streptozotocin induced diabetes in rats showed that SAC treatment exerts a therapeutic protective nature in diabetes by decreasing oxidative stress [22]. Effect of SAC on in vivo orthotopic xenograft liver tumour model demonstrated the antiproliferative and antimetastatic effects of SAC on hepatocellular carcinoma cells and suggested that SAC may be a potential therapeutic agent for treating patients with hepatocellular carcinoma [23]. Studies on the efficacy of SAC as a free radical scavenger on rat brain ischaemia models showed that SAC could have beneficial effects in brain ischaemia by the inhibition of free radical-mediated lipid peroxidation. Tests on the protective effect of SAC on carbon tetrachloride (CCl4)-induced acute liver injury in rats showed that SAC decreased CCl4-induced liver injury by attenuation of oxidative stress and proved to be a better therapeutic tool for the chronic liver disease [24].

#### 3.1.3. S-Allyl Mercapto Cysteine (SAMC)

SAMC is a biologically active and stable organosulfur compound, present in garlic. Studies of cysteine on their effects on proliferation and cell cycle progression in human colon cancer cell lines, SW-480 and HT-29 showed that SAMC potentially inhibited the growth of both cell lines. SAMC also induced apoptosis, and this was associated with an increase in caspase 3-like activity. Various reported findings suggest that SAMC is effective in colon cancer prevention [25]. Similarly, the capability of SAMC to inhibit proliferation of cancer cells was performed in two erythroleukaemia cell lines, HEL and OCIM-1, two hormone-responsive breast and prostate cancer cell lines, MCF-7 and CRL-1740, respectively, and normal human umbilical vein endothelial cells. The results provided clear evidence of the direct effect of SAMC on the established cancer cells [26]. Additionally, studies on two erythroleukaemia cell lines, HEL and OCIM-1, showed that cysteine is an effective antiproliferative agent against erythroleukaemia cells that induces cell death by apoptosis. Moreover, SAMC has shown to provide good protection against hepatotoxicity [27].

### 3.2. Oil-Soluble Sulphur Compounds

#### 3.2.1. Allicin

Allicin is a major bioactive compound obtained as a major constituent when garlic is crushed. Allicin, 2-propene-1-sulfinothioic acid S-2-propenyl ester (organosulfur), is formed when the compound alliin, (l-(+)-S-allylcysteinsulfoxide), is modified enzymatically by alliinase in garlic. These reactions happen usually when the garlic cloves are crushed. About 45 mL of allicin will be found in a garlic clove, which can be easily detected using its odour [3]. They are highly sensitive to heat and light and have the potential to easily metabolise into various compounds such as diallyl sulphides, diallyl disulphides and diallyl trisulphides. The key property of allicin is their hydrophobic nature, which makes them easily absorbed through the cell membrane without inducing any physical or chemical damage to the phospholipid bilayer, thereby it gets metabolised rapidly to exert pharmacological effects. The pharmacological effects of allicin in the cardiovascular system are highly regarded. Allicin was found to provide cardioprotective effects by inducing vasorelaxation and alleviating various pathological conditions of cardiovascular diseases, including cardiac hypertrophy, angiogenesis, platelet aggregation, hyperlipidaemia, and hyperglycaemia. Moreover, there are several other studies reporting the nature of allicin in protecting the cardiovascular system by enhancing the antioxidant status by lowering the level of reactive oxygen species and stimulating the production of glutathione [3].

Allicin has exhibited anticarcinogenic activities in a wide range of cancer cells. Allicin has been found to inhibit the cell viability of U87MG human glioma cells and induce cell death through apoptosis. This allicin-induced apoptosis was mediated through Bcl-2/Bax mitochondrial pathway, MAPK/ERK signalling pathway and antioxidant enzyme systems [28]. Study on the effect of allicin on the growth of human liver cancer Hep G2 cells showed that allicin induced p53-mediated autophagy and inhibited the viability of human hepatocellular carcinoma cell lines. Moreover, the study also revealed that allicin can potentially decrease the level of cytoplasmic p53, the PI3K/mTOR signalling pathway, and the level of Bcl-2 while they increase the expression of AMPK/TSC2 and Beclin-1 signalling pathways in Hep G2 cells. In addition, allicin-induced degradation of mitochondria was also demonstrated using confocal laser microscopy. That evidently revealed the potential of allicin as a chemopreventive agent for the prevention of liver cancer [29]. Allicin along with a few molecules of captopril, makes them a potential antidiabetic and cardiovascular protective agent for the treatment of metabolic syndrome [30].

#### 3.2.2. Diallyl Sulphides (DAS)

DAS is one of the organosulfur compounds responsible for the flavour and smell in garlic. They are lipophilic thioether derived from oxidised allicin, which is produced when garlic cloves are crushed. DAS undergo oxidation at three positions, i.e., the sulfur atom, the allylic carbon, and the terminal double bonds. DAS produces diallyl sulfoxide and diallyl sulfone by the cytochrome P450 (CYP) enzyme-mediated oxidation at the sulphur atom. These metabolites are further converted to epoxide intermediates [8]. DAS and its related compounds have inhibitory effects on chemical carcinogenesis and mutagenesis. Their content and effect on few foodborne pathogenic bacteria showed that DAS has high potential in inhibiting pathogenic bacteria [31]. Various studies have been reported on pharmacological effects of DAS. Studies showed that DAS can inhibit a wide variety of chemically induced cancers by providing protection against chemically induced hepatoxicity. Moreover, DAS hasalso reported having antibacterial, hypoglycemic, hypolipidemic and antiatherosclerotic properties.

#### 3.2.3. Diallyl Disulphides (DADS)

DADS is one of the major organosulfur compounds found in garlic oil and present in garlic cloves at 140 mM. It is found to regulate cellular proteins such as P53 and cyclin B1, induce transient cell cycle arrest in the G2/M phase and enhance the formation of reactive oxygen species in tumour cells. DADS is capable of selectively inducing redox stress in cancerous (rather than normal) cells that leads to apoptotic cell death [32]. DADS has also demonstrated their ability as a potential antioxidant agent capable of activating antioxidant enzymes such as glutathione S-transferase (GST), superoxide dismutase, catalase, etc., which further catalyse the reduction of oxidants and prevent free radical generation process [33]. Inhibition by DADS on *N*-acetyltransferase activity in *Klebsiella pneumoniae* [34], inhibition of methicillin-resistant *Staphylococcus aureus* [35] and depletion characteristics of glutathione in *Candida albicans* [36] demonstrate its antimicrobial activity. There are several human and animal studies that have been reported on the antitumor property of DADS [37]. Topical application of DAS and DADS suggests that they could effectively inhibit skin papilloma formation and significantly increase the rate of survival in the murine model [31].

#### 3.2.4. Diallyl Trisulphides (DATS)

DATS studies on diabetic rats found to improve glycemic control in them through increased insulin secretion and increased insulin sensitivity [38]. Studies of DATS on the growth of two cell lines, MCF-7 human breast cancer cells and nontumorigenic MCF-12a mammary epithelial cells, showed that DATS might offer a novel strategy for the treatment of human breast cancer [39]. DATS has found to not only alter carcinogen metabolism but also suppress the growth of cancer cells in culture and in vivo by causing cell cycle arrest and apoptosis induction [40,41].

## 4. Garlic Oil, Powder and Its Biological Uses

**Figure 2:**
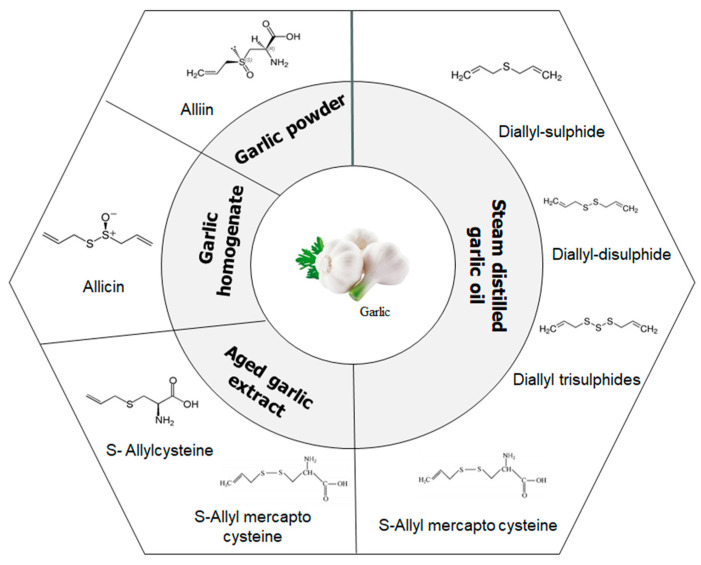
Schematic diagram of key water-soluble and oil-soluble sulphur compounds present in garlic (*A. sativum* and types of available garlic products.

Garlic powder is mainly used as a flavouring agent for condiments and processed foods. Garlic cloves are sliced or crushed, dried, and ground into powder. The composition of garlic powder is the same as that of raw garlic; however, the proportion and amount of various constituents differ significantly, whereby, the average content of alliin present in garlic is 0.8% but the raw garlic contains around 3.7 mg/gm of alliin. Crushed garlic bulb contains antimicrobial components like allicin and thiosulphate that act against *Burkholderia cepacia*, a life-threatening human pathogen [42]. In addition to fresh garlic, there are mainly four types of commercially available garlic products prepared by a variety of processing methods, including aged garlic extract, garlic oil, garlic oil macerate dehydrated and garlic powder. The medicinal uses of garlic available in the market are in the form of pills, oil and powder. Garlic oil used for therapeutic purposes is widely obtained by the steam-distillation process. Steam-distilled garlic oil contains allylmethyl, diallyl and dimethyl mono to hexa sulfide (Figure 2). The water-soluble commodities are removed from the oil. Each glove of garlic can have 0.20–0.5% of oil and contains numerous sulphide groups of DAS and DATS. The water-soluble compounds like allicin were completely removed from the garlic oil. Oil macerate products are produced usingraw garlic cloves ground in vegetable oil and are packaged in soft gel capsules. This formation includes allicin-decomposed components like sulphides, ajoene, dithiins, and residual amounts of alliin and other constituents in garlic [43].

## 5. Biological Activities of Sulfur Compounds from *Allium sativum*

**Figure 3:**
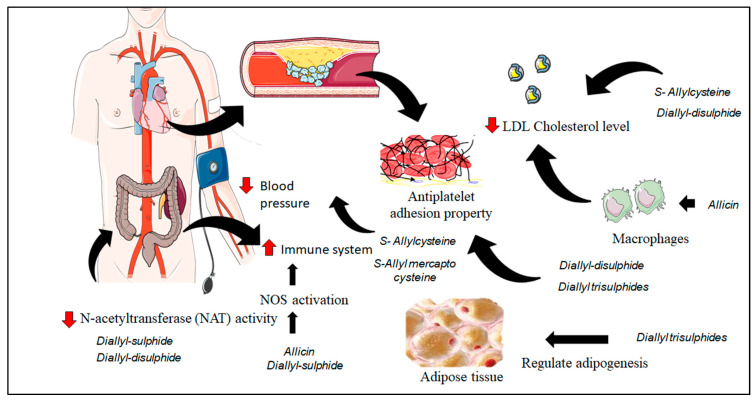
Biological activities of sulfur compounds from *A. sativum* L.

Garlic is one of the important components in people’s diet and used as herbal medicine over thousands of years [8,44]. Ancient literature reveals the importance of garlic to its health benefit towards health maintenance and treatment of diseases like heart diseases, tumours and infections (Figure 3).

### 5.1. Effects on Cardiovascular Diseases

Cardiovascular diseases are major health problem worldwide. Cardiovascular diseases (CD) are the most common and complex diseases, which are strongly associated with elevated serum levels, increased platelet activation, increased coagulation factors, increased LDL levels and alteration in glucose metabolism. The increased cholesterol and hypertension are considered to be the vital pathogenesis of cardiovascular diseases. Over the decade, much research has been materialised to develop a permanent therapeutic strategy for cardiovascular diseases. Garlic has the therapeutic potential to reduce the LDL oxidation rate and reduce the platelet aggregation results in decreasing the blood pressure. Garlic is the most common spice, which solves many health disorders as it contains many bioactive compounds including polysaccharides, saponins, and organic sulphides [45]. The sulphur content in alliums has a defence role mechanism [46]. Recent in vitro investigation revealed that water-soluble organosulfur compounds of S-allyl cysteine (SAC), and diallyl-di-sulphide (DADS) which are present in garlic extract and garlic oil respectively, are the intense inhibitors of cholesterol synthesis. The clinical uses of garlic and its component extract on cardiovascular diseases have been widely studied [47,48,49]. The water-soluble and insoluble sulphur compounds in garlic exhibits beneficial effects in cardiac abnormalities, especially for the treatment of hypercholesterolaemia and prevention of arteriosclerosis through antioxidant ability. A continuous investigation is being carried out to identify the other potent bioactive constituents in garlic. Phenolic compounds have emerged as minor but potent compounds, which are responsible for its antioxidant activity. Many studies were carried out to determine the hypercholesterolaemic action. One among them is to find out the garlic effects to reduce the amount of lipid, present in the heart patient’s blood. Groot and Scheck, in 1984, found that cholesterol and serum triglyceride level increases while taking high fat-containing meal [50]. However, later research suggested that consumption of garlic prevents the hypercholesterolemia caused by high-fat meals. A study by Bhushan et al. revealed that 10 g of fresh garlic consumed for a day for 2 months has decreased 15–28.5% of serum cholesterol levels among hypercholesterolaemic patient [51]. The inhibitory effects of garlic extract towards the cholesterol biosynthesis in hepatocytes were defined by Gebhardt in 1993. According to him, defined water-soluble components present in garlic significantly reduce the serum cholesterol level [52].

### 5.2. Effects on Atherosclerosis and Lipid Metabolism

Atherosclerosis is a complex disease, characterised by an excessive inflammatory, fibro-fatty, and proliferative response, which damages the artery walls of various cell types, specifically smooth muscle cells, monocyte-inferred macrophages, T-lymphocyte and platelets. Hyperlipidaemia is a significant pathological factor for atherosclerosis. The therapeutic benefits of garlic are well known for its lipid-lowering and antiatherogenic impacts [53,54]. DAS and DATS are the most abundant sulphur-containing volatile compounds, which are accountable for its antiatherogenic activity, mainly present inside the sleek part of the garlic. Among these constituents, allicin in the clove region is another compound that plays a crucial role in antiatherosclerotic action as well as a cancer preventive agent. Moreover, it induces the macrophages that will degrade the LDL uptake, and it can modify the lipoprotein and reduces the lipid content in the blood vessels to stop the intracellular lipid aggregation [55] A previous study exhibited that garlic reduces the atherogenic properties of cholesterol [4,6]. EGP (egg yolk-enhanced garlic powder) with copper enhances LDL oxidation in a dose-dependent manner and is used to prevent atherosclerosis [56]. The water-soluble and insoluble sulphur components present inside the garlic presents a typical adverse effect in every cardiac defect remarkably for the treatment of hypercholesterolaemia and inhibition with respect to arteriosclerosis by antioxidant ability, induction and inhibition of numerous metabolic enzymes as well via chelating activity.

### 5.3. Effects on Hypertension

Hypertension is a very significant risk factor of cardiovascular disorder. Currently, it affects 1 billion people worldwide and this number is expected to rise to 1.6 billion by 2025 [4,10]. People are using garlic as a remedy treatment to control blood pressure, worldwide. Approximately 29% of people use garlic to control their blood pressure to about 10 mmHg systolic and 8 mmHg diastolic as identical to normal blood pressure medication [51]. Hydrogen sulphide content from the garlic helps relax the blood vessels by inhibiting the angiotensin II protein [52,57]. Endothelial nitric oxide and vascular gasotransmitter hydrogen sulphide were enhanced by garlic-derived polysulfides, which induce smooth muscle cell relaxation, vasodilation and blood pressure reduction. Various dietary and genetic factors control the effectiveness of the hydrogen sulfide and nitric oxide signalling pathways that contribute to the development of hypertension. Sulfur deficiency also is part of the aetiology of hypertension and can be treated with organosulfur compounds derived from garlic. Previous studies reported that SAC from aged garlic (AG) extract acts as an antihypertensive and renoprotective agent. Day by day treatment with 600 mg of Allicor (garlic powder tablets) has proven to decrease both systolic and diastolic blood pressure. Younis, F., et al., had shown that S-allyl-mercapto-captopril (CPSSA), a conjugate of captopril with allicin, was effective in attenuating systolic and diastolic blood pressures as well as reducing blood glucose levels [30].

### 5.4. Effects on Platelets and Fibrinolytic Activity

The leading activity of the fibrinolytic system is to diffuse fibrin clots into circulation. Thrombin and plasmin are the two enzymes involved in proteolytic activities, which dissolve the insoluble fibrin protein. The inactive precursor of plasminogen can be transformed to plasmin through tissue like the plasminogen activator (tPA) or urokinase plasminogen activator (uPA). The plasmogen activator inhibitor-1 (PAI-1) as well as plasminogen activator inhibitor-2 (PAI-2), regulates the fibrinolytic activity. The fibrin accumulation in the artery can diminish the fluidity of blood circulation and induce stroke and myocardial infarction. Coumarin and warfarin remain as cost-effective prevalent medication for the fibrinolysis treatment. The enzyme derived from human urine called urokinase is employed for the thrombosis activity and it accounts low specificity to fibrin and high cost. Many medicinal plants like *Allium sativum* have various therapeutic uses including antimicrobial effect as well as fibrinolytic activities and they would effectively prevent cardiovascular diseases [58]. Garlic beneficially reduces platelet adhesion or aggregation, a potential hazard factor for cardiovascular diseases. Allicin and ajeones are pronounced to have antithrombotic activity [59]. Blood clotting is inhibited by using the garlic, which enhances the fibrinolysis action. The diallyl disulphide and diallyl trisulphide of the garlic has antiplatelet adhesion property [60]. The L-methionine from the aged garlic extract that was used as the dietary supplement by healthy individuals may be beneficial in protection against cardiovascular disease through inhibition of platelet aggregation [61]. Certain biochemical factors support and encourage the platelet aggregation together with Thromboxane A, collagen, arachidonate, adenosine diphosphate (ADP) and epinephrine. The saline and alcoholic onion extractives perfectly prohibit the aggregatory action from ADP and arachidonic acid but not thrombin in platelet-rich plasma. It has been reported that collagen, ADP, and epinephrine of platelet aggregation factors were inhibited by the consumption of garlic [62]. Catecholamines present in platelets somewhat idle against the secondary phase of ADP-induced platelet aggregation and the continuing use of garlic may suppress catecholamines involved in the thrombotic activity.

### 5.5. Effects on Cancer

Cancer is the group of diseases that can affect almost all organs of the body due to the uncontrolled and abnormal proliferation of cells. Cancer can go beyond the boundaries and start invading other parts of the body and becomes fatal for life. It is estimated that over 10 million cases are diagnosed per year worldwide [63]. As of now, several natural and synthetic compounds have been identified and tested for their anticancer properties. Garlic was reported to be a potential anticancer agent and it can modulate the various tumour mechanisms such as cell proliferation, metastasis, cell cycle, mutagenesis, and apoptosis. A study has been reported that the garlic components DAS and DADS are the effective inhibitors of bladder and colon cancer progression. The *in-vitro* study has shown that the compounds can inhibit the arylamine N-acetyltransferase (NAT) activity to produce carcinogens from foreign substances, cell growth, and DNA adduct formation of bladder and colon tumour cells in a dose-dependent manner [34,64]. Another garlic compound called allicin was reported to induce the p53 mediated autophagic cell death in Hep G2 liver cancer cells. Further, it was shown that allicin could regulate the mTOR signalling via AMPK activation and induce autophagic cell death in HepG2 cells [29]. Allicin was also reported to induce apoptosis in SKOV3 ovarian cancer cells. Mechanistically it was demonstrated that allicin stimulates the JNK pathway activation, which ultimately results in mitochondrial translocation of Bax and release of cytochrome C from mitochondria to cytosol, thus inducing the apoptosis of SKOV3 cells [65]. A subsequent study suggested that allicin might inhibit the cell viability and induce apoptosis in U87MG glioblastoma cells via regulating the Bac/Bcl2 and MAPK/ERK signalling pathways [28]. The other compound called ajoene, the organo-sulphur compound of garlic, has also been reported to induce apoptosis in the human promyeloleukaemic cell line (HL-60) through the generation of reactive oxygen species (ROS). Moreover, the induction of apoptosis and ROS generation was said to link via activation of the NF-κB signaling cascade, which is crucial for apoptosis and the ROS generation process [66]. Black garlic (BG) can treat colon cancer cells by inducing apoptosis mechanism via phosphatidylinositol 3-kinase (PI3K)/Akt pathway. Thus, garlic and its compounds are attractive therapeutic agent that can serve as an efficient anticancer agent against various cancer. Eating 10 g or more garlic in a day may significantly reduce the risk of prostate, colon, and stomach cancer. Further clinical research on garlic compounds would shed a lead-on cancer therapy

### 5.6. Effects on Immunomodulatory Response

Several synthetic and phytochemicals have been reported able to enhance the function of immune systems by stimulating the cells such as macrophages, neutrophils, dendritic cells, natural killer cells, and lymphocytes [67]. Garlic and its compounds are the crucial immunomodulators that can stimulate the immune system against various diseases. Garlic extract (allicin and DAS) were reported to reduce inflammatory bowel disease by suppressing cytokine production. The garlic extract at low concentration was shown to inhibit the production of proinflammatory cytokine (interleukin-2), and at higher concentration induces the production anti-inflammatory cytokine (interleukin-10) and seized the production of other proinflammatory cytokines such as TNF-α, IL-1α, IL-6, IL-8, IL2, and IFNγ. Thus, the inhibition of proinflammatory and induction of anti-inflammatory cytokines production led to the suppression of inflammatory bowel disease [68]. A different study has been proclaimed that garlic extract could stimulate the immune response against the *Leishmania major* infection. The mechanisms behind it were defined as that the compounds might activate the nitric oxide synthase (NOS). The NOS activation ultimately leads to the stimulation of the phagocytic activity of macrophage against *Leishmania* and eliminates it from the body [69]. In conclusion, garlic has an excellent efficacy to enhance the immune system against various diseases and act as a potent immunomodulatory substance.

### 5.7. Effects on Obesity

Obesity is a major dietary-related problem and is preventable through changing the dietary patterns. Obesity is the storage of high fat in muscles and adipose tissue, which predominantly leads to heart and vascular-related diseases. In recent times, garlic draws much attention to the treatment of obesity. Thiacremonone, a sulfur compound extracted from garlic, was reported to prevent the differentiation of adipocyte cells (3T3-L1) through the downregulation of adipogenesis associated transcription factors that are responsible for the cause of obesity [70]. Further, it was shown that treatment of thiacremonone with 3T3-L1 cells could suppress the acetyl CoA carboxylase-1 expression and increase the carnitine palmitoyltransferase via AMPK activation and led to the reduction of lipid synthesis and increase of fatty acid oxidation. DATS, the other organosulphur compound of garlic also has the potency to inhibit the differentiation of preadipocytes into adipocytes, which are an important event for obesity. DATS majorly involved in the activation of the ERK pathway and negatively regulate the adipogenic transcription factor expression during adipogenesis. These findings together suggested that garlic and its compounds could effectively reduce obesity by regulating the adipogenesis process [71].

## 6. Conclusions

Herbs are nature’s endowments to us. The usage of garlic in cultural and traditional settings had a wide range of medicinal value, which may differ from concepts accepted in current western medicine. Though garlic consumption is highly beneficial, long-term and large trials are necessary to evaluate the serious adverse effects it may pose. Consumption of raw garlic directly causes the sensation of burning in the mouth and throat or stomach, heartburn, diarrhoea, nausea and vomiting. Consumption of garlic products may cause the reddening of the gastric mucous layer and also has a chance of having hyphaemia leading to permanent vision loss. Sometimes it might increase the risk of bleeding including nosebleeds and bleeding gums. In some individuals, consumption of excess garlic may also cause GERD (gastroesophageal reflux disease) and loss of appetite. Overdose of garlic may lead to kidney haematoma and had a chance of getting an autoimmune disorder of pemphigus. The safe dose of garlic consumption should be tested particularly in pregnant or breastfeeding women and also in young children. During the second and third trimesters of pregnancy, it is advisable to take a moderate level of garlic cloves to lower blood pressure and maintain good development of the baby, however consuming large quantities of garlic during pregnancy may lead to some adverse effect such as blood thinning and miscarriage. The U.S Food and Drug Administration, certified garlic as “generally recognized as safe” (GRAS) for consuming garlic as a flavouring agent as well as food including during lactation. Some reports stated that working with garlic could cause side effects like asthma and possible allergic reactions. may lead to miscarriages. However, it is advisable to consult the well-trained practitioner for using the herbal supplements.

## Figures and Tables

**Table 1:** Structure and chemical formula of the sulphur based compounds present in garlic.

Compound	Structure	Molecular Formula
**Water-Soluble Sulphur Compounds**		
Alliin	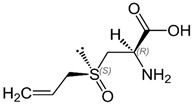	C_6_H_11_NO_3_S
S- Allylcysteine	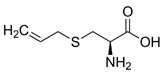	C_6_H_11_NO_2_S
S-Allyl mercapto cysteine	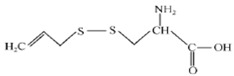	C_6_H_11_NO_2_S_2_
**Oil-Soluble Sulphur Compounds**		
Allicin	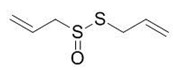	C_6_H_10_OS_2_
Diallyl sulphides	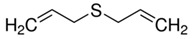	C_6_H_10_S
Diallyl disulphides	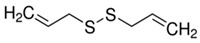	C_6_H_10_S_2_
Diallyl trisulphides	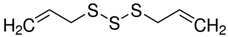	C_6_H_10_S_3_

## References

[1] Banerjee S.K., Maulik S.K. (2002). Effect of garlic on cardiovascular disorders: A review. Nutr. J..

[2] Zahid Ashraf M., Hussain M.E., Fahim M. (2005). Antiatherosclerotic effects of dietary supplementations of garlic and turmeric: Restoration of endothelial function in rats. Life Sci..

[3] Chan J.Y.Y., Yuen A.C.Y., Chan R.Y.K., Chan S.W. (2013). A review of the cardiovascular benefits and antioxidant properties of allicin. Phyther. Res..

[4] Kearney P.M., Whelton M., Reynolds K., Muntner P., Whelton P.K., He J. (2005). Global burden of hypertension: Analysis of worldwide data. Lancet.

[5] Gonen A., Harats D., Rabinkov A., Miron T., Mirelman D., Wilchek M., Weiner L., Ulman E., Levkovitz H., Ben-Shushan D. (2006). The antiatherogenic effect of allicin: Possible mode of action. Pathobiology.

[6] Mag P., Bose S., Laha B., Banerjee S. (2014). Quantification of allicin by high performance liquid chromatography ultraviolet analysis with effect of post ultrasonic sound and microwave radiation on fresh garlic cloves. Pharmacog. Mag..

[7] Kimura S., Tung Y.C., Pan M.H., Su N.W., Lai Y.J., Cheng K.C. (2017). Black garlic: A critical review of its production, bioactivity, and application. J. Food Drug Anal..

[8] Mikaili P., Maadirad S., Moloudizargari M., Aghajanshakeri S., Sarahroodi S. (2013). Therapeutic uses and pharmacological properties of garlic, shallot, and their biologically active compounds. Iran J. Basic Med. Sci..

[9] Rose P., Moore P.K., Whiteman M., Zhu Y.Z. (2019). An appraisal of developments in allium sulfur chemistry: Expanding the pharmacopeia of garlic. Molecules.

[10] Fukushima S., Takada N., Wanibuchi H., Hori T., Min W., Ogawa M. (2001). Recent advances on the nutritional effects associated with the use of garlic as a supplement suppression of chemical carcinogenesis by water-soluble organosulfur. J. Nutr..

[11] Chobanian A.V., Bakris G.L., Black H.R., Cushman W.C., Green L.A., Izzo J.L., Jones D.W., Materson B.J., Oparil S., Wright J.T. (2003). Seventh report of the Joint National Committee on prevention, detection, evaluation, and treatment of high blood pressure. Hypertension.

[12] Kumar K.P.S., Bhowmik D., Chiranjib, Tiwari P., Khare R. (2010). *Allium sativum* and its health benefits: An overview. J. Chem. Pharm. Res..

[13] Bhandari P.R. (2012). Garlic (Allium sativum L.): A review of potential therapeutic applications. Int. J. Green Pharm..

[14] Lawson L.D., Wang Z.J. (2005). Allicin and allicin-derived garlic compounds increase breath acetone through allyl methyl sulfide: Use in measuring allicin bioavailability. J. Agric. Food Chem..

[15] Batiha G.E., Beshbishy A.M., Wasef L.G. (2020). Chemical constituents and pharmacological activities of garlic (*Allium*
*sativum* L.): A Review. Nutrients.

[16] Zhang Y., Liu X., Ruan J., Zhuang X., Zhang X., Li Z. (2020). Phytochemicals of garlic: Promising candidates for cancer therapy. Biomed. Pharmacother..

[17] Londhe V.P., Gavasane A.T., Nipate S.S., Bandawane D.D., Chaudhari P.D. (2011). Role of garlic (*Allium*
*sativum*) in various diseases: An overview. J. Pharma.Res..

[18] Bordia A., Verma S.K., Srivastava K.C. (1998). Effect of garlic (*Allium*
*sativum*) on blood lipids, blood sugar, fibrinogen and fibrinolytic activity in patients with coronary artery disease. Prostaglandins Leukot Essent Fatty Acids..

[19] Kim K.M., Chun S.B., Koo M.S., Choi W.J., Kim T.W., Kwon Y.G., Chung H.T., Billiar T.R., Kim Y.M. (2001). Differential regulation of NO availability from macrophages and endothelial cells by the garlic component S-allyl cysteine. Free Radic. Biol. Med..

[20] Welch C., Wuarin L., Side N. (1992). Antiproliferative effect of the garlic compound on human neuroblastoma cells in vitro S-ally1 cysteine. Cancer Lett..

[21] Ashafaq M., Khan M.M., Shadab Raza S., Ahmad A., Khuwaja G., Javed H., Khan A., Islam F., Siddiqui M.S., Safhi M.M. (2012). S-allyl cysteine mitigates oxidative damage and improves neurologic deficit in a rat model of focal cerebral ischemia. Nutr. Res..

[22] Saravanan G., Ponmurugan P. (2010). Beneficial effect of S-allylcysteine (SAC) on blood glucose and pancreatic antioxidant system in streptozotocin diabetic rats. Plant Foods Hum. Nutr..

[23] Ng K.T.P., Guo D.Y., Cheng Q., Geng W., Ling C.C., Li C.X., Liu X.B., Ma Y.Y., Lo C.M., Poon R.T.P. (2012). A garlic derivative, s-allylcysteine (sac), suppresses proliferation and metastasis of hepatocellular carcinoma. PLoS ONE.

[24] Numagami Y., Ohnishi S.T. (2001). S-Allylcysteine Inhibits Free Radical Production, Lipid Peroxidation and Neuronal Damage in Rat Brain Ischemia. Molecules.

[25] Shirin H., Pinto J.T., Kawabata Y., Soh J., Delohery T., Moss S.F., Murty V., Rivlin R.S., Holt P.R., Weinstein I.B. (2001). Antiproliferative effects of S-allylmercaptocysteine on colon cancer cells when tested alone or in combination with sulindac sulfide. Cancer Res..

[26] Sigounas G., Hooker J.L., Li W., Anagnostou A., Sigounas G., Hooker J.L., Li W., Anagnostou A., Steiner M. (2009). S-Allylmercaptocysteine, a stable thioallyl compound, induces apoptosis in erythroleukemia cell lines. Nutr. Cancer.

[27] Sumioka I., Matsura T., Yamada K. (2001). Therapeutic effect of S-allylmercaptocysteine on acetaminophen-induced liver injury in mice. Eur. J. Pharmacol..

[28] Cha J.H., Choi Y.J., Cha S.H., Choi C.H., Cho W.H. (2012). Allicin inhibits cell growth and induces apoptosis in U87MG human glioblastoma cells through an ERK-dependent pathway. Oncol. Rep..

[29] Chu Y.L., Ho C.T., Chung J.G., Rajasekaran R., Sheen L.Y. (2012). Allicin induces p53-mediated autophagy in Hep G2 human liver cancer cells. J. Agric. Food Chem..

[30] Younis F., Mirelman D., Rabinkov A., Rosenthal T. (2010). S-allyl-mercapto-captopril: A novel compound in the treatment of cohen-rosenthal diabetic hypertensive rats. J. Clin. Hypertens..

[31] Murillo G., Mehta R.G. (2005). Chemoprevention of chemically-induced mammary and colon carcinogenesis by 1alpha-hydroxyvitamin D5. Steroid Biochem. Mol. Biol..

[32] Altonsy M.O., Andrews S.C. (2011). Diallyl disulphide, a beneficial component of garlic oil, causes a redistribution of cell-cycle growth phases, induces apoptosis, and enhances butyrate-induced apoptosis in colorectal adenocarcinoma cells (HT-29). Nutr. Cancer.

[33] Dhuley J.N., Naik S.R., Rele S., Banerji A. (1999). Hypolipidaemic and antioxidant activity of diallyl disulphide in rats. Pharm. Pharmacol. Commun..

[34] Chen G.W., Chung J.G., Hsieh C.L., Lin J.G. (1998). Effects of the garlic components diallyl sulfide and diallyl disulfide on arylamine N-acetyltransferase activity in human colon tumour cells. Food Chem. Toxicol..

[35] Tsao S., Hsu C., Yin M. (2003). Garlic extract and two diallyl sulphides inhibit methicillin-resistant *Staphylococcus aureus* infection in BALB/cA mice. J. Antimicrob. Chemother..

[36] Laadan B., Almeida J.R., Rådström P., Hahn-Hägerdal B., Gorwa-Grauslund M. (2008). Identification of an NADH-dependent 5-hydroxymethylfurfural-reducing alcohol dehydrogenase in *Saccharomyces cerevisiae*. Yeast.

[37] Yi L.V., So K.F., Wong N.K., Xiao J. (2019). Anti-cancer activities of S-allylmercaptocysteine from aged garlic. Chin. J. Nat Med..

[38] Liu C., Hse H., Lii C., Chen P., Sheen L. (2005). Effects of garlic oil and diallyl trisulfide on glycemic control in diabetic rats. Eur. J. Pharm..

[39] Malki A., El-saadani M., Sultan A.S. (2009). Garlic constituent diallyl trisulfide induced apoptosis in MCF7 human breast cancer cells. Cancer Biol. Ther..

[40] Powolny A.A., Singh S.V. (2008). Multitargeted prevention and therapy of cancer by diallyl trisulfide and related Allium vegetable-derived organosulfur compounds. Cancer Lett..

[41] Seki T., Hosono T., Hosono-fukao T. (2008). Anticancer effects of diallyl trisulfide derived from garlic. Asia Pac. J. Clin. Nutr..

[42] Wallock-Richards D., Doherty C.J., Doherty L., Clarke D.J., Place M., Govan J.R.W., Campopiano D.J. (2014). Garlic revisited: Antimicrobial activity of allicin-containing garlic extracts against *Burkholderia cepacia* complex. PLoS ONE.

[43] Zeng Y.W., Sun D., Du J., Pu X.Y., Yang S.M., Yang X.M., Yang T., Yang J.Z. (2016). Identification of QTLs for resistant starch and total alkaloid content in brown and polished rice. Genet. Mol. Res..

[44] Gebhardt R. (1993). Multiple inhibitory effects of garlic extracts on cholesterol biosynthesis in hepatocytes. Lipids.

[45] Lee H.S., Lim W.C., Lee S.J., Lee S.H., Lee J.H., Cho H.Y. (2016). Antiobesity effect of garlic extract fermented by *Lactobacillus plantarum* BL2 in diet-induced obese mice. J. Med. Food.

[46] Nwachukwu I.D., Slusarenko A.J., Gruhlke M.C.H. (2012). Sulfur and sulfur compounds in plant defence. Nat. Prod. Commun..

[47] Rietz B., Isensee H., Strobach H., Makdessi S., Jacob R. (1993). Cardioprotective actions of wild garlic (*Allium*
*ursinum*) in ischemia and reperfusion. Mol. Cell. Biochem..

[48] Kendler B.S. (1987). Garlic (*Allium*
*sativum*) and onion (*Allium*
*cepa*): A review of their relationship to cardiovascular disease. Prev. Med..

[49] Koscielny J., Klüßendorf D., Latza R., Schmitt R., Radtke H., Siegel G., Kiesewetter H. (1999). The antiatherosclerotic effect of *Allium sativum*. Atherosclerosis.

[50] Liu J., Zhang G., Cong X., Wen C. (2018). Black garlic improves heart function in patients with coronary heart disease by improving circulating antioxidant levels. Front. Physiol..

[51] Osamor P.E., Owumi B.E. (2010). Complementary and alternative medicine in the management of hypertension in an urban Nigerian community. BMC Complement Altern Med..

[52] Benavides G.A., Squadrito G.L., Mills R.W., Patel H.D., Isbell T.S., Patel R.P., Darley-Usmar V.M., Doeller J.E., Kraus D.W. (2007). Hydrogen sulfide mediates the vasoactivity of garlic. Proc. Natl. Acad. Sci. USA.

[53] Schwartz C.J., Valente A.J., Sprague E.A. (1993). A modern view of atherogenesis. Am. J. Cardiol..

[54] Obenin I.A., Myasoedova V.A., Iltchuk M.I., Zhang D.W., Orekhov A.N. (2019). Therapeutic effects of garlic in cardiovascular atherosclerotic disease. Chin J. Nat. Med..

[55] Bayan L., Koulivand P.H., Gorji A. (2014). Garlic: A review of potential therapeutic effects. Avicenna J Phytomed..

[56] Yamaji K., Sarker K.P., Abeyama K., Maruyama I. (2004). Anti-atherogenic effects of an egg yolk-enriched garlic supplement. Int. J. Food Sci. Nutr..

[57] Banerjee S.K., Mukherjee P.K., Maulik S.K. (2003). Garlic as an antioxidant: The good, the bad and the ugly. Phyther. Res..

[58] Ansari F., Soltan M.N., Naderi G., Sadegh S.M., Karimi A. (2011). Original article study of garlic effect on fibrinolytic activity of the blood clot in vitro. Iran. J. Pediatr. Hematol. Oncol..

[59] Teranishi K., Sc R., Romano E., Dkc C., Robson S.C., Teranishi K., Apitz-Castro R., Robson S.C., Romano E., Cooper D.K.C. (2003). Inhibition of baboon platelet aggregation in vitro and in vivo by the garlic derivative, ajoene. Xenotransplantation.

[60] Aslani N., Entezari M.H., Askari G., Maghsoudi Z., Maracy M.R. (2016). Effect of garlic and lemon juice mixture on lipid profile and some cardiovascular risk factors in people 30–60 years old with moderate hyperlipidaemia: A randomized clinical trial. Int. J. Prev. Med..

[61] Rahman K. (2007). Effects of garlic on platelet biochemistry and physiology. Mol. Nutr. Food Res..

[62] Ackermann R.T., Mulrow C.D., Ramirez G., Gardner C.D., Morbidoni L., Lawrence V.A. (2001). Garlic shows promise for improving some cardiovascular risk factors. Arch. Intern. Med..

[63] Eaton L. (2003). World cancer rates set to double by 2020. BMJ.

[64] Lin J.G., Chen G.W., Su C.C., Hung C.F., Yang C.C., Lee J.H., Chung J.G. (2002). Effects of garlic components diallyl sulfide and diallyl disulfide on arylamine N-acetyltransferase activity and 2-aminofluorene-DNA adducts in human promyelocytic leukemia cells. Am. J. Chin. Med..

[65] Xu L., Yu J., Zhai D., Zhang D., Shen W., Bai L., Cai Z., Yu C. (2014). Role of JNK activation and mitochondrial Bax translocation in allicin-induced apoptosis in human ovarian cancer SKOV3 cells. Evidence-based Complement. Altern. Med..

[66] Dirsch V.M., Gerbes A.L., Vollmar A.M. (1998). Ajoene, a compound of garlic, induces apoptosis in human promyeloleukemic cells, accompanied by generation of reactive oxygen species and activation of nuclear factor κB. Mol. Pharmacol..

[67] Patil U.S., Jaydeokar A.V., Bandawane D.D. (2012). Immunomodulators: A pharmacological review. Int. J. Pharm. Pharm. Sci..

[68] Hodge G., Hodge S., Han P. (2002). *Allium sativum* (garlic) suppresses leukocyte inflammatory cytokine production in vitro: Potential therapeutic use in the treatment of inflammatory bowel disease. Cytometry.

[69] Ghazanfari T., Hassan Z.M., Khamesipour A. (2006). Enhancement of peritoneal macrophage phagocytic activity against Leishmania major by garlic (*Allium*
*sativum*) treatment. J. Ethnopharmacol..

[70] Arreola R., Quintero-Fabián S., López-Roa R.I., Flores-Gutiérrez E.O., Reyes-Grajeda J.P., Carrera-Quintanar L., Ortuño-Sahagún D. (2015). Immunomodulation and anti-inflammatory effects of garlic compounds. J. Immunol. Res..

[71] Yang C., Li L., Yang L., Lǚ H., Wang S., Sun G. (2018). Anti-obesity and Hypolipidemic effects of garlic oil and onion oil in rats fed a high-fat diet. Nutr. Metab..

